# Oestradiol: any role in cardiovascular risk factors in female centenarians of Hainan?

**DOI:** 10.1186/s12872-019-1103-6

**Published:** 2019-05-16

**Authors:** Qiao Zhu, Yao Yao, Chao-Xue Ning, Xiao-Ping Chen, Fu-Xin Luan, Liang Liu, Qiong Liu, Na Wang, Fu Zhang, Ya-Li Zhao

**Affiliations:** 1 Hainan branch of PLA General Hospital, Sanya, 572000 China; 2 Center for Healthy Aging and Development Studies, National School of Development, Peking University, Beijing, China

**Keywords:** Centenarians, Women, Oestradiol, Cardiovascular disease, Dyslipidemia

## Abstract

**Background:**

Previous studies reported that low level of oestradiol (E2) was associated with higher risk of cardiovascular disease (CVD). However, little study examined the relationship between E2 and CVD in longevous women, which were deficient in serum E2 for the post-menopausal status. Therefore, this study aims to explore the association between E2 and CVD risk factors in a group of female centenarians of Hainan, China.

**Methods:**

A total of 413 female centenarians (aged from 100 to 115) from China Hainan Centenarian Cohort Study (CHCCS) were enrolled in this study. Home interviews were conducted to collected data on demographic characteristics, health-related lifestyles, and anthropometrics. The level of serum E2 was assessed in the Clinical Laboratory of Hainan branch of PLA General Hospital. The variables of CVD risk factors, including blood pressures, lipids and blood glucose, were measured through standard procedures.

**Results:**

Significant negative correlations between levels of serum E2 and TC, HDL-C, and LDL-C were observed in this study. Compared with the highest group of E2, the odds ratio and 95% confidence intervals of high LDL-C in groups 3, 2 and 1 were OR1.94 (CI0.82–4.62), OR3.61 (CI1.27–10.25) and OR9.29 (CI2.08–41.53), respectively. Similarly, the prevalence of hypertension was decreased with the increase of serum E2. The odds ratio and 95% confidence intervals of stage-2 hypertension in groups 3, 2 and 1 versus highest E2 group were OR1.34 (CI0.49–3.72), OR1.36 (CI0.47–3.99) and OR1.38 (CI0.45–4.20), respectively.

**Conclusions:**

This study examined the relationship between E2 and CVD risk factors in a group of community-based female centenarians. A negative correlations between serum E2 levels and CVD risk factors, i.e. high level of LDL-C, TC, and hypertension were observed in this population. Besides, the level of serum E2 is also negatively correlated with HDL-C. Further studies on the correlation between serum E2 and CVD risk factors, especially dyslipidemia, in longevous and post-menopausal women are warranted.

## Background

The number of Chinese centenarians, according to the data from China CENSUS, has increased from 17,877 to 35,954 between 2000 and 2010. And Hainan province has the fastest growth rate of centenarians, with an increase of 254% from 3.78 /100,000 to 13.4/100,000. Currently, studies suggested that centenarians are a template for healthy aging to cope with global ageing and steadily improve their life expectancy [1, 2].

Historical demographic data from 37 countries revealed that women live longer than men. In industrialized countries, gender gap in life expectancy ranges from approximately 4 to 10 years. The gap may be related to sex hormones [3]. Moreover, there are significant differences in sex hormone levels between pre-menopausal and postmenopausal women, and the incidence of CVD in postmenopausal women is significantly increased [4]. This suggests that sex hormones play an important role in the development of CVD. Actually, CVD is one of the most serious causes of death in the elderly. Epidemiological studies have shown that sex hormones are associated with CVD risk factors, such as BP, TC, TG, HDL-C and LDL-C. However, there are few studies on female centenarians.

Hainan province is an independent island. Due to historical and economic factors, centenarians are indigenous, and show a natural aging process. This study explored the relationship between E2 and CVD risk factors among female centenarians in Hainan, providing basic data and theoretical support for healthy aging.

## Methods

### Methods

A cross-sectional study was conducted between June 2014 and March 2016. We investigated 413 female centenarians from 18 regions throughout Hainan province. All are native settlers.

The surveyors were strictly trained before investigation, and the survey content, operative procedure and methods were standardized. Height (H), weight (W), waist circumference (WC) and hip circumference (HC) were measured with participants dressed in light clothing and barefoot. We measured the height and weight of the elderly with a scale (Seca, Germany). Each parameter was measured twice. We computed the BMI and WHR using the following standard formula: BMI=W/H^2^, WHR = WC/HC.

In the sitting position, we measured the blood pressure of the centenarians two times by electronic sphygmomanometers (Omron Hem-7200, Japan). At least 1-min intervals between the measurements, and the reported blood pressures were the average of the two measurements. If the difference between the first and second measurement was more than 5 mmHg, the repeated measurements were performed.

Samples of venous blood were extracted from the centenarians and transported within 4 h in chilled bio-transport containers (4 °C) to clinical laboratory. The estradiol III kit (Roche) was used to detect fresh serum estradiol levels in female centenarians by electrochemiluminescence (Cobas E602). The lower detection threshold of the kit was 18.4 pmol/L. The fresh plasma TC, HDL-C and LDL-C levels were measured by enzyme colorimetry (Cobas C701) using Cholesterol Gen.2 kit, HDL-Cholesterol plus 3rd generation kit, and LDL-Cholesterol Gen.3 kit respectively. The content of fresh plasma TG was determined by colorimetry (Cobas C701) using Triglycerides kit (Roche). Fresh plasma glucose was examined by hexokinase method (Glucose HK Gen.3 kit, Cobas C701).

### Definition of CVD risk factors

The 2017 Hypertension Clinical Practice Guidelines provided a new categorization of BP levels. This guideline defined BP categories of normal, elevated, or stage 1 or 2 hypertension. SBP/DBP thresholds of 130/80 mmHg now define the diagnosis of “ stage 1 hypertension”, and 140/90 mmHg define the diagnosis of “stage 2 hypertension” [5]. As for plasma glucose, we use the reference range provided in the reagent instructions, 4.16–6.72 mmol/L (> 90 years old) (Glucose HK Gen.3 kit, Roche Diagnostics, North America).

The cut-off points for dyslipidemia were plasma TC ≥ 240 mg/dl and/or use of medications to lower blood cholesterol for high TC, TG ≥ 200 mg/dl for high TG, HDL-C<40 mg/dl for low HDL-C, and LDL-C ≥ 160 mg/dl and/or use of medications to lower blood cholesterol for high LDL-C.

### Ethical approval

The study protocol was approved by the ethical committee of the Hainan branch of PLA General Hospital (Sanya, China). Each participant provided written informed consent to be included in the study.

### Data management

All paper-based information was stored in the data cabinet and managed by specific personnel. The data validity was checked by hand on a unified model. Then, we used Epidata 3.0 software to enter the data twice, and SPSS 19.0 software (SPSS Inc., Chicago, IL, USA) was used for statistical analysis.

### Statistical analysis

Descriptive data were shown as the mean ± SD. By multiple linear regression analysis, we analyzed the relationship between E2 and physical examination adjusted for BMI, WC, WHR, SBP, DBP, glucose, TC, TG, HDL-C and LDL-C. With the increase of variables, we presented a cumulative R2. Multiple logistic regression was used to evaluate the association between E2 and CVD risk factors adjusted for age, BMI, WC, WHR and the number of children. A *P*-value< 0.05 was considered statistically significant.

## Results

### General information of female centenarians

A total of 413 female centenarians completed the survey. We collected basic information from the standardized questionnaires. The results showed that all participants were married, and 97.1% of centenarians engaged in physical labour. 55.7% of the elderly lived in the countryside, while 44.3% in the urban area. As for the number of children, 2.4% of the subjects had no children, 9.7% had one child and 87.9% had two or more children. Also, we measured the E2 and CVD risk factors, including body measurement variables (BMI, WC, WHR, SBP and DBP), blood glucose and lipids (TC, TG, HDL-C and LDL-C) in Table 1.

**Table 1:** Characteristics of Study Subjects

Variables	*N* = 413	Reference Range
Age (mean ± SD)	102.8 ± 2.9	–
E2(mean ± SD)	46.99 ± 28.86	40-100 pmol/L
BMI (mean ± SD)	17.69 ± 3.67	18.5–22.9
WC (mean ± SD)	75.13 ± 8.95	<80 cm
WHR (mean ± SD)	0.90 ± 0.08	<0.8
SBP (mean ± SD)	154.46 ± 24.77	< 130 mmHg
DBP (mean ± SD)	75.12 ± 13.02	< 80 mmHg
glucose (mean ± SD)	5.15 ± 1.43	4.16–6.72 mmol/L(> 90 years old)
TC (mean ± SD)	185.86 ± 37.54	< 200 mg/dl
TG (mean ± SD)	106.39 ± 61.87	< 150 mg/dl
HDL-C (mean ± SD)	56.89 ± 14.83	> 40 mg/dl
LDL-C (mean ± SD)	112.07 ± 30.84	< 130 mg/dl

### The relationship between E2 and physical examination

**Fig. 1:**
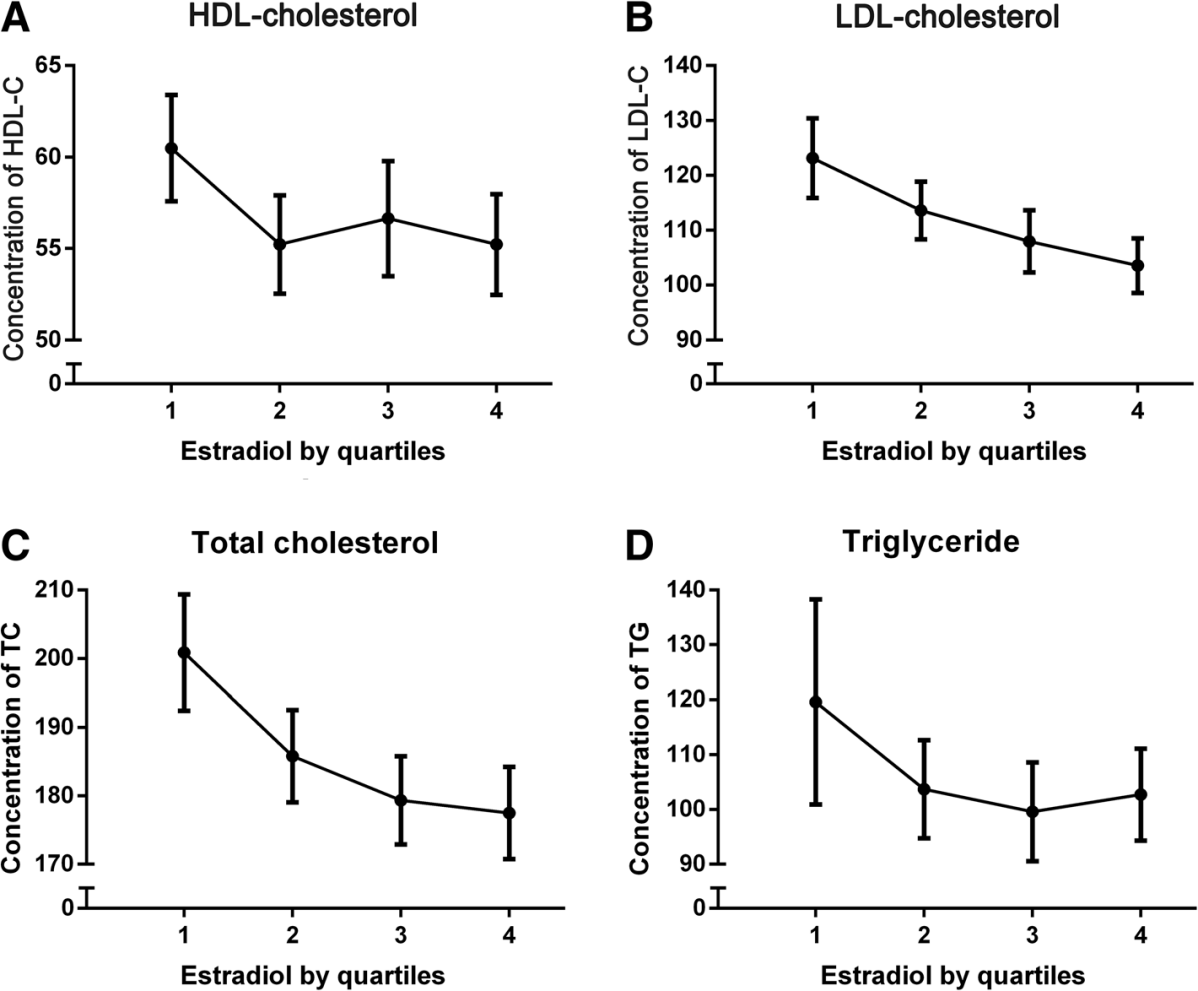
Concentration of TC, TG, HDL-C, and LDL-C according to estradiol by quartiles. The concentrations of TC, TG, HDL-C, LDL-C were demonstrated as mean and 95%CI according to quartiles of estradiol (1st quartile: ≤19.4, 2nd quartile: 19.5–40.3, 3rd quartile: 40.4–62.9, 4th quartile:≥63.0)

By multiple linear regression analysis, we have found significant negative correlation between levels of E2 and TC, HDL-C, LDL-C, and no significant correlation between E2 and either SBP or DBP and also TG. However, after adjusting for BMI, WC, WHR, SBP, DBP, glucose, TC, TG, HDL-C and LDL-C, there was a positive correlation between E2 and TC (Table 2). As the number of variables increased, R2 gradually rose, indicating that the model was getting better. Furthermore, according to the level of E2, quartiles are used for grouping, named 1, 2, 3 and 4, respectively. It have shown significant negative correlation between E2 and TC, LDL-C (Fig. 1).

**Table 2:** Multiple Linear Regression between E2 and Physical Examination

variables	beta	95%CI	*P*	adjusted beta	95%CI	*P*	R^2^
BMI	− 0.097	−1.521~ − 0.003	0.049	− 0.122	−1.855~ − 0.060	0.037	0.009
WC	− 0.014	− 0.358~0.267	0.775	0.156	0.009~0.995	0.046	0.011
WHR	−0.051	−50.196~15.738	0.305	−0.137	− 92.177~ − 0.922	0.046	0.018
SBP	−0.096	−0.224~0.000	0.051	−0.047	− 0.189~0.080	0.427	0.025
DBP	−0.066	−0.361~0.068	0.179	−0.014	− 0.289~0.225	0.808	0.026
Glucose	−0.033	−2.630~1.290	0.502	−0.045	−2.829~1.025	0.358	0.028
TC	−0.205	−0.231~ − 0.085	0.000	0.449	0.060–0.631	0.018	0.061
TG	−0.045	−0.066~0.024	0.357	−0.107	− 0.105~0.006	0.078	0.061
HDL-C	−0.154	−0.486~ − 0.113	0.002	− 0.321	−0.933~ − 0.316	0.000	0.073
LDL-C	−0.198	−0.275~ − 0.097	0.000	− 0.566	−0.845~ − 0.215	0.001	0.098

Adjusted for BMI, WC, WHR, SBP, DBP, concentrations of non-fast blood glucose, TC, TG, HDL-C and LDL-C. The first beta is the unadjusted values. E2 is used as a continuous variable in the model

### The association between E2 and CVD risk factors

Multiple logistic regressions was used to evaluate the association between E2 and CVD risk factors adjusted for age, BMI, WC, WHR and the number of children, as shown in Table 3. Compared with the highest group of E2, the odds ratio and 95% confidence intervals of high LDL-C in groups 3, 2 and 1 were OR1.94 (CI0.82–4.62), OR3.61 (CI1.27–10.25) and OR9.29 (CI2.08–41.53), respectively. Similarly, the prevalence of hypertension was decreased with the increase of serum E2. The odds ratio and 95% confidence intervals of stage-2 hypertension in groups 3, 2 and 1 versus highest E2 group were OR1.34 (CI0.49–3.72), OR1.36 (CI0.47–3.99) and OR1.38 (CI0.45–4.20), respectively. And, high TC also showed a similar trend. However, it should be noted that there was no centenarians with high TC in the group 1. So, the data in this column was absence. There were no significant correlation between E2 and the incidence of high TG, low HDL.

**Table 3:** Multiple Logistic Regression between E2 and CVD Risk Factors

Variable		1st quartile	2nd quartile	3rd quartile	4th quartile
		OR (95%CI)	OR (95%CI)	OR (95%CI)	OR (95%CI)
Hypertension					
Stage 1	Unadjusted	1.18 (0.51–2.72)	0.95 (0.42–2.13)	0.66 (0.31–1.42)	1.00
	Adjusted	1.13 (0.48–2.64)	0.85 (0.37–1.96)	0.62 (0.28–1.38)	1.00
Stage 2	Unadjusted	1.38 (0.45–4.20)	1.36 (0.47–3.99)	1.34 (0.49–3.72)	1.00
	Adjusted	1.31 (0.42–4.08)	1.23 (0.41–3.69)	1.21 (0.43–3.46)	1.00
High TC ^a^	Unadjusted	–	5.60 (1.83–17.10)^**^	2.39 (1.03–5.56)^*^	1.00
	Adjusted	–	5.75 (1.85–17.83)^**^	2.34 (0.99–5.50)	1.00
High TG	Unadjusted	1.43 (0.44–4.66)	3.68 (0.75–18.17)	1.82 (0.52–6.43)	1.00
	Adjusted	1.51 (0.43–5.31)	3.88 (0.74–20.48)	1.71 (0.45–6.55)	1.00
Low HDL	Unadjusted	1.88 (0.61–5.81)	3.08 (1.07–8.91)^**^	2.80 (0.96–8.16)	1.00
	Adjusted	2.17 (0.68–6.96)	3.27 (1.10–9.71)^**^	3.17 (1.05–9.51)^**^	1.00
High LDL	Unadjusted	9.29 (2.08–41.53)^**^	3.61 (1.27–10.25)^**^	1.94 (0.82–4.62)	1.00
	Adjusted	10.22 (2.22–46.98)^**^	3.63 (1.25–10.51)^**^	1.83 (0.76–4.43)	1.00

The prevalence of hypertension, high level of TC, TG, LDL-C and low level of HDL-C was demonstrated according to quartiles of estradiol

The cut-off points for dyslipidemia were plasma TC ≥ 240 mg/dl and/or use of medications to lower blood cholesterol for high TC, TG ≥ 200 mg/dl for high TG, HDL-C<40 mg/dl for low HDL-C, and LDL-C ≥ 160 mg/dl and/or use of medications to lower blood cholesterol for high LDL-C

According to the level of E2, quartiles are used for grouping (1st quartile:≥19.4,2nd quartile:19.5–40.3, 3rd quartile:40.4–62.9 4th quartile:≥63.0) The odds ratios were presented as unadjusted and further adjusted for age, BMI, WC, WHR and the number of children

^a^ No centenarian with concentration of TC higher than 240 mg/dl in the first quartile thus the data was unavailable in this column

*P < 0.05, **P < 0.01

## Discussion

Human longevity has an obvious tendency of maternal inheritance, and the average life expectancy of women in the world is higher than that of men [6]. With acceleration of China’s population aging, the proportion of elderly women is increasing. Centenarian is a model of exceptional longevity [7]. So, we explored the relationship between E2 and cardiovascular risk factors through a study of 413 female centenarians in Hainan. Our results showed centenarians presented light weight [8], partly because of aging, which causes the structure and function decline of the organism, and partly because of E2, which reduces with ovarian recession and has an impact on glucose and lipid metabolism [9].

CVD is one of the leading causes of death worldwide, and it is more prevalent in postmenopausal women. Our results suggested that E2 was negatively correlated with LDL-C. E2 binds to receptors on hepatocytes, activates enzymes that affect lipid metabolism, promotes absorption of residual LDL by up-regulated LDL receptors, and accelerates the conversion of hepatic cholesterol to bile acids [10]. Moreover, E2, as a radical scavenger, is able to break the formation of free radical chains generated by the membrane oxidation processes, thereby inhibiting LDL and VLDL oxidation [11, 12]. Additionally, E2 can decrease the susceptibility of LDL-C to LDL oxidation, prevent the oxidative damage and protect the endothelial [13]. Also, we have found the incidence of high LDL-C decreased with the increase of E2. Consistent with previous studies, this suggests that E2 has a direct effect on LDL metabolism [14, 15]. It may be that the reduction of E2 leads to a decrease in postmenopausal LDL receptors [10].

Interestingly, we also found significant negative correlation between levels of E2 and HDL-C. HDL is considered to be a protective factor for atherosclerosis, helping to degrade cholesterol from VLDL and chylomicron by reverse the transport of cholesterol from peripheral tissues to the liver. E2 can regulate the rate of synthesis of HDL structural apolipoprotein in the liver. It increases HDL by enhancing the rate of apoA-I and apoA-II synthesis [16]. There is huge discrepancy in the level of HDL in postmenopausal women. Some studies have shown a significant decrease in HDL levels in postmenopausal women, while some studies have shown a significant increase in HDL levels [10, 17]. The result of this difference may be due to variation in the study population, life interventions, and duration of menopause. On the other hand, HDL has different types of subtypes. Studies showed that the severity of CHD was correlated with the distribution of HDL subtypes. Large particles of HDL2b significantly decreased, while small particles of preβ1-HDL and HDL3 increased [18–20]. The level of the HDL3 subtype in female postmenopausal women is higher than that before menopause, suggesting that sex hormones may affect the distribution of HDL subtypes, which may be related to the rapid increase in the CVD risk in women after menopause. Furthermore, the study on HDL in the eldest Jews and their offspring in northern Europe also demonstrated that the diameter of HDL particles was larger, indicating that genetic factors influence the distribution of HDL subtypes [21]. However, we did not test the subtype of HDL, which is a limitation of this study. We need to perform further studies to understand the role of HDL in female centenarians.

Hypertension is one of the major risk factors of CVD. Our result indicated the incidence of hypertension decreased with the increase of E2, especially in stage 2 hypertension. Studies have found that E2 has multiple protective effects on the cardiovascular system [22, 23]. Our results are agreement with these studies. This is because E2 can inhibit vasoconstriction in response to acetylcholine as well as reduce the level of endothelin to dilate vessels [24]. Also, E2 can improve blood rheology through the nitric oxide pathway and play a protective role in blood vessels [25]. Studies have shown that E2 can directly act on vascular EC to enhance NO production through both genomic stimulation of eNOS expression [26, 27] and membrane receptor-mediated, non-genomic activation of the enzymatic activity. Furthermore, E2 may increase the release of PGI 2, a potent inhibitor of platelet aggregation and strong vasodilator [28]. In addition, E2 increases the production of endothelium-derived hyperpolarizing factor (EDHF), which activates K+ channels, causes hyperpolarization, and in turn inhibits Ca2+ influx and causes VSM relaxation [29].

### Limitations of the study

Due to number of subjects, the generalizability of the results is limited. Second, we did not test the subtype of HDL, which is a limitation of this study. We need to perform further studies to understand the role of HDL in female centenarians. Third, the study is only a cross-sectional study. We can not rule out the possibility of withdrawal bias in the annual follow-up of this large sample of centenarians.

## Conclusion

This study examined the relationship between E2 and CVD risk factors in a group of community-based female centenarians. A negative correlations between serum E2 levels and CVD risk factors, i.e. high level of LDL-C, TC, and hypertension were observed in this population. Besides, the level of serum E2 is also negatively correlated with HDL-C. Further studies on the correlation between serum E2 and CVD risk factors, especially dyslipidemia, in longevous and post-menopausal women were warranted.

## Abbreviations

BMI: Body mass index
CVD: Cardiovascular disease
DBP: Diastolic blood pressure
E2: Oestradiol
HC: Hip circumference
HDL-C: High-density lipoprotein cholesterol
LDL-C: Low-density lipoprotein cholesterol
SBP: Systolic blood pressure
TC: Total cholesterol
TG: Triglyceride
WC: Waist circumference
WHR: Waist-hip ratio
